# Correlation of Fc Receptor Polymorphisms with Pneumococcal Antibodies in Vaccinated Kidney Transplant Recipients

**DOI:** 10.3390/vaccines10050725

**Published:** 2022-05-05

**Authors:** Marie-Luise Arnold, Falko M. Heinemann, Simon Oesterreich, Benjamin Wilde, Anja Gäckler, David Goldblatt, Bernd M. Spriewald, Peter A. Horn, Oliver Witzke, Monika Lindemann

**Affiliations:** 1 Department of Internal Medicine, Friedrich-Alexander University Erlangen-Nürnberg, Krankenhausstraße 12, 91054 Erlangen, Germany; marlies.arnold@web.de (M.-L.A.); bernd.spriewald@uk-erlangen.de (B.M.S.); 2 Institute for Transfusion Medicine, University Hospital, Virchowstraße 179, 45147 Essen, Germany; falko.heinemann@uk-essen.de (F.M.H.); simon.oesterreich@gmx.de (S.O.); peter.horn@uk-essen.de (P.A.H.); 3 Department of Nephrology, University Hospital Essen, Hufelandstraße 55, 45147 Essen, Germany; benjamin.wilde@uk-essen.de (B.W.); anja.gaeckler@uk-essen.de (A.G.); 4 Department of Infectious Diseases, West German Centre of Infectious Diseases, University Hospital Essen, Hufelandstraße 55, 45147 Essen, Germany; oliver.witzke@uk-essen.de; 5 World Health Organization Reference Laboratory for Pneumococcal Serology Based at the Great Ormond Street Institute of Child Health, University College London, 30 Guilford Street, London WC1N 1 EH, UK; d.goldblatt@ucl.ac.uk; 6 Great Ormond Street Children’s Hospital NHS Foundation Trust, Great Ormond Street, London WC1N 3JH, UK

**Keywords:** Fcγ receptor polymorphism, Fcα receptor polymorphism, *Streptococcus pneumoniae*, vaccination, kidney transplantation, pneumococcal antibodies, serotype-specific opsonophagocytic function, HLA antibodies

## Abstract

Several polymorphisms within Fc receptors (FCR) have been described, some of which correlate with allograft function. In the current study, we determined three Fcγ receptor and five Fcα receptor dimorphisms in 47 kidney transplant recipients who had been vaccinated against *Streptococcus pneumoniae*. We analyzed if FCR genotypes correlated with pneumococcal antibodies and their serotype-specific opsonophagocytic function, tested prior to and at months 1 and 12 post-vaccination. In parallel, we assessed antibodies against HLA and MICA and determined kidney function. We observed that IgG2 antibodies against pneumococci at months 1 and 12 after vaccination and IgA antibodies at month 1 differed significantly between the carriers of the three genotypes of FCGR3A rs396991 (V158F, *p* = 0.02; 0.04 and 0.009, respectively). Moreover, the genotype of FCGR3A correlated with serotype-specific opsonophagocytic function, reaching statistical significance (*p* < 0.05) at month 1 for 9/13 serotypes and at month 12 for 6/13 serotypes. Heterozygotes for FCGR3A had the lowest antibody response after pneumococcal vaccination. On the contrary, heterozygotes tended to have more antibodies against HLA class I and impaired kidney function. Taken together, our current data indicate that heterozygosity for FCGR3A may be unfavorable in kidney transplant recipients.

## 1. Introduction

Infection and rejection are two major obstacles in transplantation medicine. Both are mediated by humoral and cellular immune responses, which are suppressed after transplantation. Antibodies against microorganisms and allografts bind to Fc receptors and can thereby lead to immune activation. A polymorphism within the Fcγ receptor (FCGR), FCGR2A rs1801274 [1], is correlated with the recurrence of acute otitis media after infection with *Streptococcus pneumoniae* (*S. pneumoniae*) [2] (Table 1). *S. pneumoniae* is a gram positive bacterium that frequently colonizes the human nasopharynx [3]. Outside the nasopharynx, it can cause lobar pneumonia, meningitis, otitis media, or sinusitis, and it is especially harmful after coinfection with the influenza virus [4]. A severe form of infection is invasive pneumococcal disease (IPD), which has a fatality rate of approximately 10% [3,5,6]. According to data by the Centers for Disease Control and Prevention, the rate of IPD in organ transplant recipients is 25 times greater than in the general population [5,6]. Vaccination against *S. pneumoniae* is recommended in individuals with immunocompromising conditions because it has been shown to reduce the incidence of IPD [7,8,9].

There is already significant data on the associations between FCGR polymorphisms and transplant outcome [10,11,12,13,14]. Three FCGR polymorphisms may be predictive of graft survival, rejection, and HLA antibodies after solid organ transplantation or of immune recovery after hematopoietic stem cell transplantation (FCGR2A rs1801274 [10,11], FCGR3A rs396991 [15], and FCGR3B rs35139848 [16]) (Table 1).

**Table 1:** Previous functional data on Fcγ receptor polymorphisms.

Fc Receptor	SNP (Substitution)	Amino Acid	Function	Reference
FcγRIIa	FCGR2A	H131R	R131 homozygous:	
	rs1801274 (A vs. G)		Recurrence of acute otitis media after infection with *Streptococcus pneumoniae* ↑	[2]
			Graft survival ↓	[10]
			Acute rejections ↑	[11]
FcγRIIIa	FCGR3A	V158F	Affects the affinity of IgG1 to IgG4	[17,18,19,20,21]
	rs396991		and influences immune cell activation	
	(G vs. A)		V158 carriers:	
			Peritubular capillaritis ↑	[15]
			IFN-γ ↑ after HLA antibody stimulation	
FcγRIIIb	FCGR3B	(Intron)	Neutrophil antigen resulting in the	[17,19]
	rs35139848		isoform neutrophil antigen 1 (NA1) vs.	
	(A vs. G)		NA2, affecting N-linked glycosylation	
			of the FcγR	
			Influences neutrophil recovery, severe	[16]
			infections, and transplant-related	
			mortality after hematopoietic stem	
			cell transplantation	

SNP—single nucleotide polymorphism.

In previous studies on kidney transplant recipients, we determined pneumococcal immunity prior to and at months 1 and 12 after vaccination with the conjugate vaccine Prevenar (Prevenar 13^®^, Pfizer, New York, NY, USA). We measured antibodies of the IgG, IgG2, and IgA isotypes and the serotype-specific antibody function by an opsonophagocytic-killing assay (OPA) [22]. In parallel, we assessed IgG antibodies against human leukocyte antigens (HLA) and major histocompatibility complex class I chain-related antigens A (MICA) [23], as it has been debated that vaccination may induce HLA antibodies and allograft rejection [24,25,26,27,28,29,30]. We observed that kidney transplant recipients could mount a significant response of pneumococcal antibodies after vaccination, though at a lower level than healthy controls [22]. We furthermore found that in females, but not in males, non-specific HLA-antibodies (i.e., those without donor specificity) increased after vaccination [23]. Apart from IgG antibodies, IgA antibodies against HLA may impact allograft survival [31]. FcαR (CD89) is also capable of triggering IgA-mediated immune responses to pathogens, and it has been proposed to function in circulating IgA clearance [32]. The FcαR gene (FCAR) also contains polymorphic positions.

In the current study, we tested 47 kidney transplant recipients for three FCGR and five FCAR dimorphisms. We analyzed if the respective genotypes correlated with pneumococcal antibodies, with HLA antibodies, or with allograft function.

## 2. Materials and Methods

### 2.1. Patients

In total, 47 previously described [23], clinically stable kidney transplant recipients (18 female, 29 male) with a median age of 53 years (range 21–73 years) were included in this prospective, single center study. In addition to pneumococcal antibodies [22] and HLA/MICA antibodies [23], we tested for three FCGR and five FCAR dimorphisms. All patients received a single dose of Prevenar. Blood was drawn immediately prior to vaccination and at months 1 and 12 after vaccination. The median interval between kidney transplantation and vaccination was 49 months (i.e., 4.1 years; range 4 months to 34 years). The median follow-up after vaccination was 51 months (range 44–57 months). The median serum creatinine concentration (range) was 1.7 (0.9–4.9), 1.7 (1.0–5.1), and 1.6 (0.9–4.4) mg/dL pre-vaccination, at month 1, and month 12, respectively, corresponding to an estimated glomerular filtration rate (eGFR) of 42 (13–83), 40 (12–84), and 46 (11–83) mL/min/1.73 m^2^, respectively (as determined by the Chronic Kidney Disease Epidemiology Collaboration formula [33]). Ten patients were treated with cyclosporine A, 31 with tacrolimus, 34 with mycophenolate mofetil (MMF), 44 with prednisone, four with everolimus, and two with eculizumab. Thus, the majority was treated with tacrolimus, MMF, and prednisone. This study was approved by the institutional review board of the University Hospital Essen (14-5858-BO) and written informed consent was obtained from all participants. It was carried out in accordance with the Declarations of Helsinki and Istanbul and their latter amendments.

### 2.2. Vaccine

The 13-valent pneumococcal vaccine Prevenar contains the polysaccharides of 13 pneumococcal serotypes (1, 3, 4, 5, 6A, 6B, 7F, 9V, 14, 18C, 19A, 19F, and 23F), individually conjugated to a nontoxic mutant form of diphtheria toxin cross-reactive material 197 (CRM197).

### 2.3. Genotyping of FCAR and FCGR Polymorphisms

The genotyping of three FCGR polymorphisms (FCGRIIA (rs1801274, 519A/G, codon 131 histidin to arginine), FCGRIIIA (rs396991, 559G/A, codon 158 valin to phenylalanin) and FCGRIIIB (rs35139848, neutrophil antigen 1/2 (NA1)/NA2)), four intron polymorphisms (rs10402324, rs11084377, rs1865097, and rs4806608), and one exon 5 polymorphism (rs16986050, 844A/G, which changes codon 248 from AGC (Serin) to GGC (Glycin) in the cytoplasmic domain of the receptor) of the FCAR on chromosome 19 was carried out in a StepOnePlus real-time PCR detection system (Applied Biosystems, Darmstadt, Germany) using TaqMan SNP Genotyping Assay and TaqMan Universal PCR Master Mix, No AmpErase UNG (Applied Biosystems, Darmstadt, Germany). A TaqMan MGB probe labelled with VICTM dye detected allele 1 and a probe labelled with FAMTM dye detected allele 2.

### 2.4. Determination of Antibodies against Pneumococci

Antibodies against *S. pneumoniae* were determined by three ELISA formats which detect IgG, IgG2, or IgA antibodies against 23 pneumococcal serotypes (VaccZyme™, The Binding Site, Schwetzingen, Germany) [22]. The assay was performed according to the manufacturer’s instructions.

Moreover, the serotype-specific functional response was measured by a validated killing assay (OPA), as published previously [22,34]. Briefly, heat-inactivated sera were serially diluted. Target bacteria were added to assay plates and incubated for 30 min at 25 °C. Baby rabbit complement (3–4 weeks old, Pel-Freez Biologicals, Rogers, AR, USA, 12.5% final concentration) and differentiated HL-60 cells (ATCC CCL-240) [34,35] were then added to each well at an approximate effector-to-target ratio of 200:1 or 400:1, depending on serotypes. These HL-60 cells were differentiated into granulocytes using 100 mM dimethylformamide (DMF). The viability of the differentiated HL-60 cells was assessed by trypan blue exclusion and annexin V/propidium iodide staining. The HL-60 cells were incubated with DMF for at least 3–4 days and used only if CD35 (complement receptor 1) expression was up-regulated by ≥ 55% of the cell population and CD71 (transferrin receptor) expression was down-regulated by ≤ 15% of the cell population, as assessed by flow cytometry. Moreover, HL-60 cells express low levels of FcγRIII (CD16). Unfortunately, we have no information on the FCGRIIIA V158F polymorphism; however, it is known that HL-60 cells are homozygous for the arginine R131 allele of FCGRIIA [36]. The assay plates with effector and target cells were incubated for 45 min at 37 °C on a shaker. Thereafter, the reaction was stopped and a 10 µL aliquot was transferred to each well of a MultiScreen HTS HV filter plate (Millipore, Livingston, UK), applying vacuum. Then 150 µL of HySoy medium was added to each well and filtered through. The filter plates were incubated at 37 °C with 5% CO2 overnight, then fixed with Destain Solution (Bio-Rad, Watford, UK). Thereafter, the plates were stained with Coomassie Blue and destained once. Colonies were enumerated on a Cellular Technology Limited (CTL) ImmunoSpot Analyzer^®^ (Cleveland, OH, USA). The OPA is generally accepted as the best functional correlate of pneumococcal immunity [34,37]. The interpolated reciprocal serum dilution that resulted in complement-mediated killing of 50% of the bacteria was defined as the OPA titer. The lower limit of quantitation (LLOQ) was 1:8 and was determined during assay validation for each serotype. Values below the LLOQ were defined as half of the LLOQ (1:4). Serum samples were tested by OPA at the Pfizer Vaccines Research Laboratory (New York, NY, USA) for all 13 serotypes included in the Prevenar vaccine.

### 2.5. Determination of HLA and MICA Antibodies

All samples were tested for IgG antibodies against HLA class I and class II and MICA using Luminex™ technology-based assays (LABScreen™ Mixed Beads, One Lambda/Thermo Fisher, Canoga Park, CA, USA) according to the manufacturer’s instructions and as described in detail previously [23]. In brief, the responses towards each bead were scored as 8 (positive), 4 (undetermined), or 1 (negative), and the respective responses were summed up. Thereby, we obtained antibody score values for HLA class I and class II and MICA of 12-96, 5-40, and 2-16, respectively.

### 2.6. Statistical Analysis

Data were analyzed using GraphPad Prism version 8.4.2 for Windows (GraphPad Prism Software, La Jolla, CA, USA) or IBM SPSS Statistics version 23 (Armonk, NY, USA). The correlation of FCR genotypes with pneumococcal or HLA and MICA antibodies was analyzed by the Kruskal–Wallis test with Dunn’s multiple comparisons test. The correlation of FCR genotypes with allograft function or with immunosuppressive treatment was analyzed by contingency tables (chi-square test or Fisher’s exact test, as appropriate). Moreover, a Bonferroni correction for multiple testing was applied. As we tested for eight independent FcR polymorphisms, we multiplied the *p* values by eight. The results were considered significant at *p* < 0.05.

## 3. Results

### 3.1. Correlation between Fc Receptor Polymorphisms and Pneumococcal Antibodies

**Figure 1:**
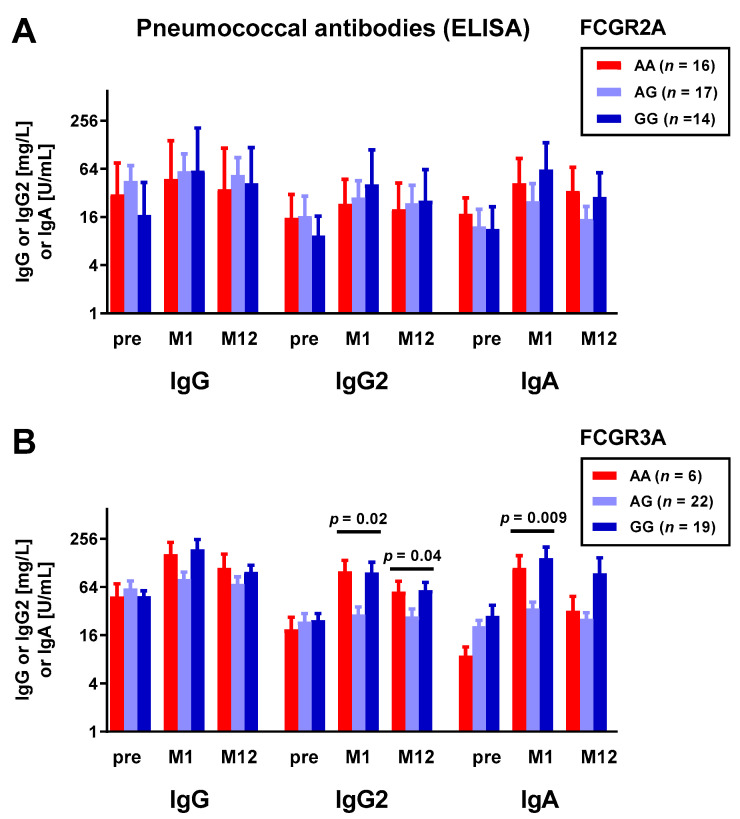
Kidney transplant recipients were genotyped for the polymorphisms FCGR2A rs1801274 (**A**) and FCGR3A rs396991 (**B**). IgG, IgG2, and IgA antibodies determined by commercial ELISA (VaccZyme™) prior to pneumococcal vaccination (pre) and at month 1 (M1) and month 12 (M12) thereafter were grouped by genotype. Antibodies are shown on a logarithmic scale (log2) as geometric means and geometric standard deviation factors [39]. The three groups were compared by Kruskal–Wallis test with Dunn’s multiple comparisons test. Of note, none of the results remained significant after Bonferroni correction.

We determined the concentration of IgG, IgG2, and IgA antibodies against *S. pneumoniae* pre- and post-vaccination. Based on the observation that especially soluble FcRs present immunomodulatory properties, for example, the inhibition of B cell proliferation and immunoglobulin production [21,38], we grouped the pneumococcal antibodies by eight FCR genotypes and compared the respective groups of clinically stable kidney transplant recipients by Kruskal–Wallis test. We assessed three time points: prior to vaccination, at month 1, and at month 12. Whereas findings on the FCGR2A polymorphism (FcγRIIa H131R) showed no significant result (Figure 1A), the findings on the FCGR3A polymorphism (FcγRIIIa V158F) reached statistical significance (Figure 1B). IgG2 antibodies against pneumococci at months 1 and 12 after vaccination differed significantly between the groups (*p =* 0.02 and *p =* 0.04), and heterozygous carriers of FCGR3A (genotype AG) had the lowest antibody concentration. Similarly, the IgA antibodies against pneumococci at month 1 differed significantly (*p =* 0.009). However, after Bonferroni correction for multiple testing, none of the results remained significant. Of note, pneumococcal antibodies prior to vaccination did not differ significantly between the three genotype groups.

**Figure 2:**
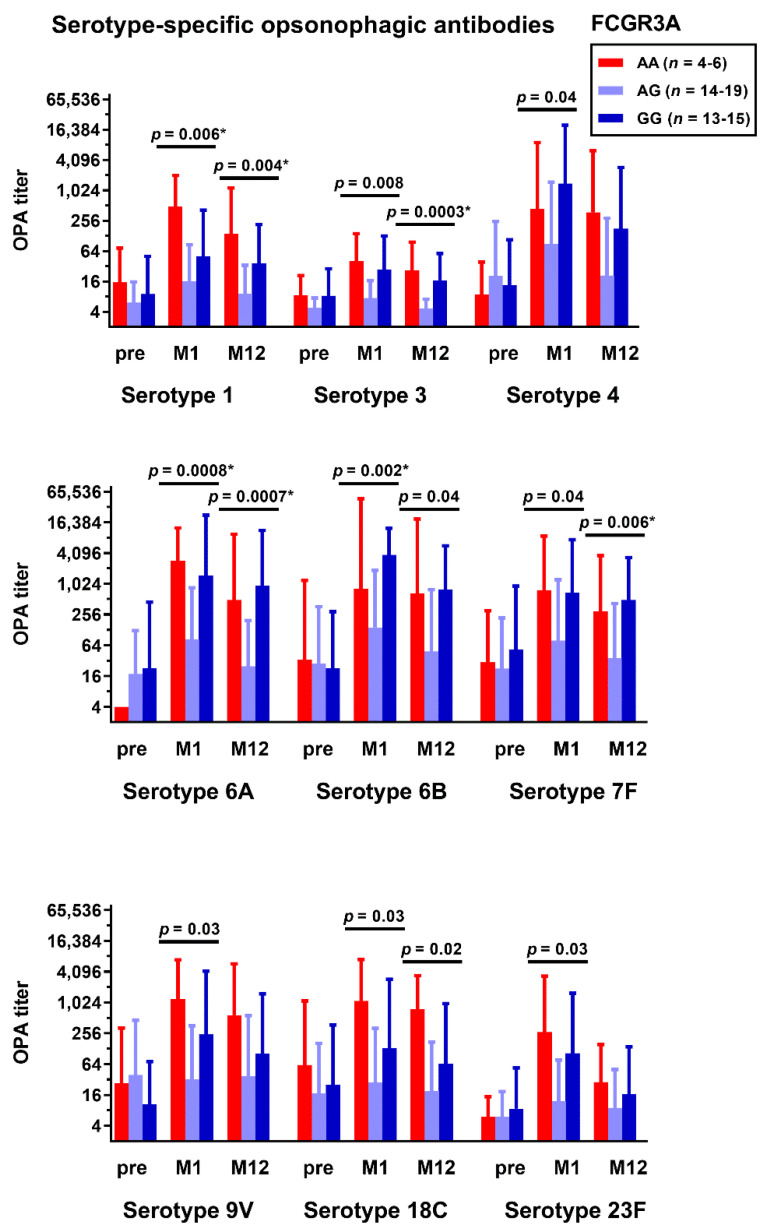
Kidney transplant recipients were genotyped for the FCGR3A rs396991 polymorphism and the serotype-specific opsonophagocytic antibody (OPA) responses, as determined prior to pneumococcal vaccination (pre) and at month 1 (M1) and month 12 (M12) thereafter. The recipients were grouped by genotype. OPA titers are shown on a logarithmic scale (log2) as geometric mean titers and geometric standard deviation factors [39]. The three groups were compared by Kruskal–Wallis test with Dunn’s multiple comparisons test. The results that remained significant after Bonferroni correction are marked with an asterisk.

We furthermore considered antibody function as determined by serotype-specific OPA. This assay is a measure not only of the antibody concentrations, but also of their binding to Fc receptors. Similar to the ELISA data, serotype-specific OPA results were significantly dependent on the FCGR3A genotype (Figure 2). In detail, differences between genotypes at month 1 after vaccination reached statistical significance (*p <* 0.05) in 9 out of 13 serotypes tested (1, 3, 4, 6A, 6B, 7F, 9V, 18C, and 23F). At month 12, differences between genotypes were still significant for 6 out of 13 serotypes (1, 3, 6A, 6B, 7F, and 18C). After Bonferroni correction, at month 1, three results remained significant (for serotype 1, 6A, and 6B), and at month 12, four remained significant (for serotype 1, 3 6A, and 7F). The remaining FCAR and FCGR polymorphisms did not correlate significantly with the pneumococcal antibodies. Moreover, after vaccination, the heterozygous carriers of FCGR3A (genotype AG) showed only a minor increase in pneumococcal antibody concentration and function, in contrast to the homozygous carriers (AA or GG genotypes).

**Figure 3:**
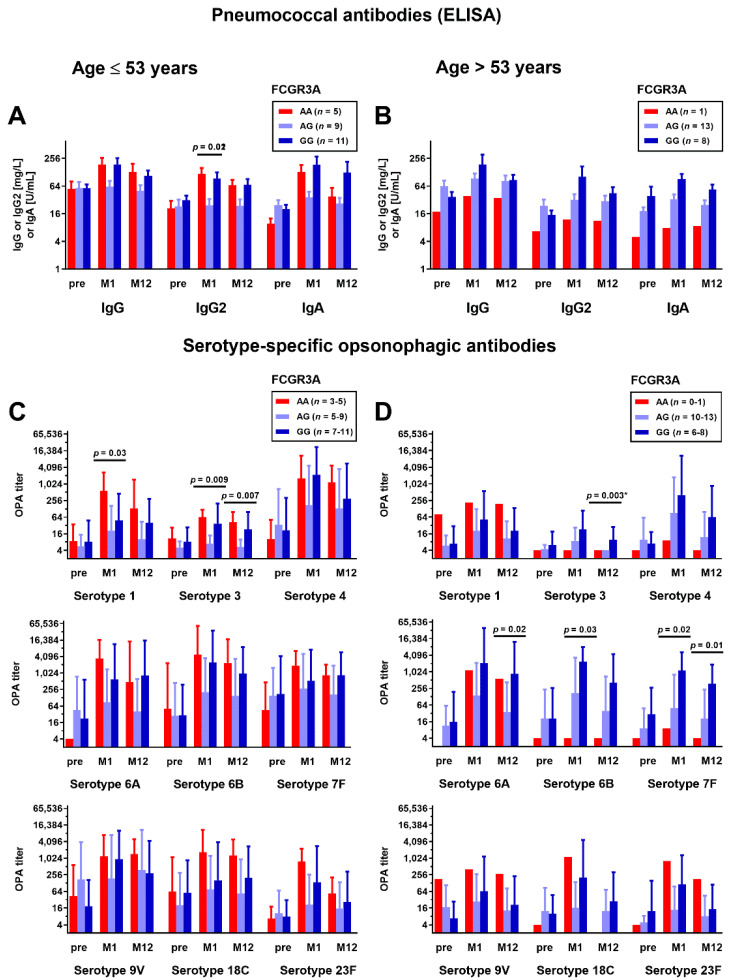
Kidney transplant recipients were genotyped for the FCGR3A rs396991 polymorphism and antibodies determined by commercial ELISA (**A**,**B**), and serotype-specific opsonophagocytic antibody (OPA) responses (**C**,**D**) were divided by genotype and age group. The left panels show data in younger patients (≤ median age of 53 years), while the right panels show data in older patients (>53 years). Antibodies were determined prior to pneumococcal vaccination (pre) and at month 1 (M1) and month 12 (M12) thereafter. The results are shown on a logarithmic scale (log2) as geometric means and geometric standard deviation factors [39]. The three genotype groups were compared by Kruskal–Wallis test with Dunn’s multiple comparisons test. The results that remained significant after Bonferroni correction are marked with an asterisk.

As there is a known dysregulation of the immune system and functional decline in antibody function with aging, we analyzed our patient cohort separately by age (median 53 years). Overall, data on FCGR3A were similar in younger and older patients, as depicted in Figure 3. In detail, pneumococcal antibodies as determined by ELISA were lower in carriers of the AG vs. GG genotype of FCGR3A, independent of age (Figure 3A,B). As the older cohort comprised only one patient with the genotype AA, a further comparison was not possible. Moreover, the OPA data indicated that carriers of the AG vs. GG genotype had weaker responses after vaccination in both age groups (Figure 3C,D).

### 3.2. Correlation between Fc Receptor Polymorphisms and HLA and MICA Antibodies

**Figure 4:**
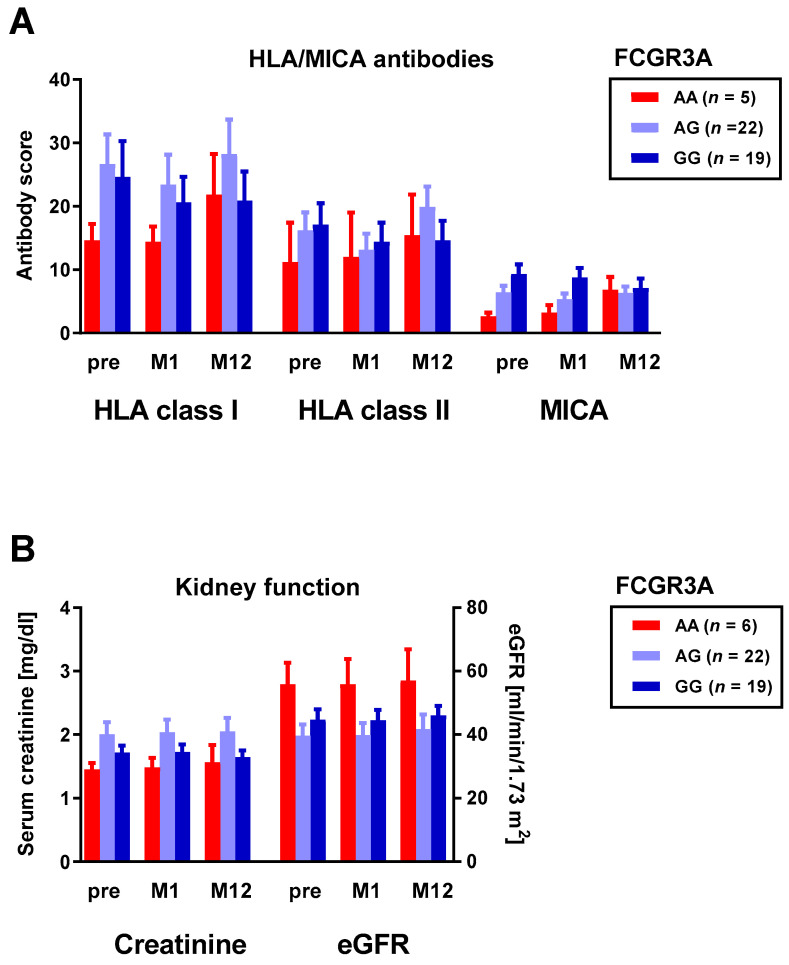
Kidney transplant recipients were genotyped for the FCGR3A rs396991 polymorphism and antibodies and kidney function were determined prior to pneumococcal vaccination (pre) and at month 1 (M1) and month 12 (M12) thereafter. Panel (**A**) shows antibodies against human leukocyte antigens (HLA) and major histocompatibility class I-related chain A (MICA). HLA/MICA antibodies were determined by Luminex™ technology. Responses to individual Luminex™ beads were summed up and an antibody score was generated, as detailed in Section 2. Panel (**B**) indicates serum creatinine concentration and estimated glomerular filtration rate (eGFR). The data represent means and standard errors of the mean (SEM).

We grouped HLA and MICA antibodies by FCR genotype and compared the respective groups of renal transplant recipients at three time points. The antibody score was used as a measure of the strength of the HLA and MICA antibodies [23], and eight FCR polymorphisms were considered. Using the Kruskal–Wallis test, we observed that HLA and MICA antibody responses did not differ significantly between the carriers of the respective FCR genotypes. However, we observed that the carriers of the AG genotype of FCGR3A tended to show the highest antibody scores for HLA class I (Figure 4A), which fits with impaired allograft function (Figure 4B). This phenomenon occurred irrespective of the time point (both pre- and post-pneumococcal-vaccination).

### 3.3. Correlation between Fc Receptor Polymorphisms and Kidney Fucntion

**Figure 5:**
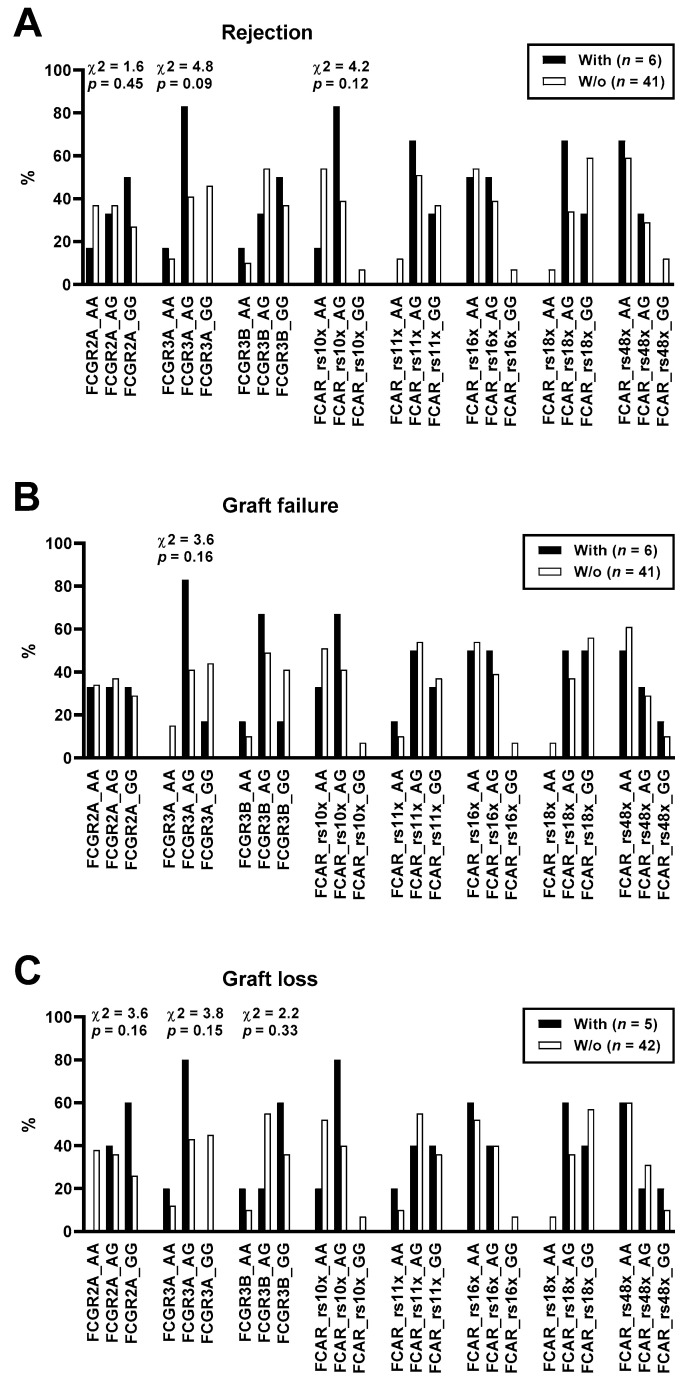
Genotype frequencies of Fcα and Fcγ receptor polymorphisms (FCAR and FCGR, respectively) in 47 kidney transplant recipients with vs. without (w/o) rejection (**A**), graft failure (**B**), and graft loss (**C**). Using a chi-square test, we compared genotype frequencies (AA, AG, and GG). 2A—rs1801274; 3A—rs396991; 3B—rs35139848; rs10x—rs10402324; rs11x—rs11084377; rs16x—rs16986050; rs18x—rs1865097; rs48x—rs4806608.

We also analyzed if eight FCR polymorphisms correlated with allograft rejection, failure, and loss. Using a chi-square test, we compared genotype frequencies (AA, AG, and GG) in patients with and without the respective events (Figure 5). We observed that in patients with allograft rejection, the genotype GG of FCGR2A, the genotype AG of FCGR3A, and the genotype AG of FCAR rs10402324 were most abundant (χ^2^ = 1.6, 4.8, and 4.2, respectively) (Figure 5A). The frequency of genotype AG of FCGR3A was also enhanced in patients with allograft failure and loss (χ^2^ = 3.6 and 3.8, respectively) (Figure 5B,C). Moreover, in patients with allograft loss, the genotype GG of FCGR2A and the genotype GG of FCGRB3B were found more frequently (χ^2^ = 3.6 and 2.2, respectively) (Figure 5C). However, most likely because only six patients suffered from rejection and five from graft loss, none of the findings reached statistical significance.

Furthermore, we analyzed the data set by Fisher’s exact test, applying a dominant and recessive model. We observed that in carriers of the (dominant) A allele of FCGR2A, allograft rejection and loss occurred less frequently. When using a dominant model (AA vs. AG plus GG) we found an odds ratio (OR) of 0.6 and 0.0, respectively (Table 2). Moreover, carriers of the GG genotype of FCGR3A suffered less frequently from rejection, graft failure, and loss. Using a dominant model, differences in the frequency of rejection and graft loss just escaped statistical significance (*p* = 0.07 for both parameters). Of note, both values for the OR were 0.0, pointing to a protective effect of the GG genotype. The frequency of the AG genotype of FCGR3A was doubled in patients with vs. without rejection, graft failure, and loss. Taken together, data on FCGR3A were most consistent and showed by trend that the genotype AG may correlate with impaired kidney function.

### 3.4. Correlation between Fc Receptor Polymorphisms and Immunosuppressive Treatment

After performing Fisher’s exact test for the eight FCR polymorphisms and the presence or absence of immunosuppressive drugs as described in the Methods section, only one result reached statistical significance (FCGR2A and cyclosporine A: *p* = 0.03 prior to correction for multiple testing, non-significant after correction). Six out of sixteen patients with the genotype AA of FCGR2A received cyclosporine A, as did four out of seventeen with AG and zero out of fourteen with GG. Nevertheless, carriers of the AA and GG genotypes of FCGR2A showed similar levels of pneumococcal antibodies. However, differences in allograft rejection or graft loss with between the FCGR2A genotype groups could, in principle, be caused by treatment with cyclosporine A. Only six patients suffered from rejection and five from allograft loss, of which three had the genotype GG. Of note, there was no correlation between the FCGR3A polymorphism and immunosuppressive treatment. Due to this small number, the statistical analysis needs to be interpreted with caution.

### 3.5. Correlation between Kidney Function and Pneumococcal Antibodies

We used the serum creatinine concentration or eGFR as markers of kidney function. Spearman analysis of either serum creatinine or eGFR showed no significant correlation with IgG, IgG2, or IgA antibodies against pneumococci. But–as expected–correlation coefficients with serum creatinine were negative at months 1 and 12 after vaccination (mean values of −0.08 and −0.12, respectively), i.e., lower serum creatinine correlated with higher antibody concentrations. Accordingly, correlation coefficients with eGFR were positive. A similar trend was observed for the OPA results.

## 4. Discussion

The current study indicates that the genotype AG of FCGR3A rs396991 (FcγRIIIa-V158F) may be unfavorable in kidney transplant recipients. It correlated with significantly lower antibody responses after pneumococcal vaccination and also with an impaired functional capacity of the antibodies, which may cause more infectious complications. Because the HL-60 cell line with its Fc receptors—which remain to be further defined—is the same in all OPA assays in our current study, we hypothesize that the FCGR3A polymorphism correlates with different concentrations of pneumococcal antibodies. The OPA does not include cells with patient-specific FcγR IIIa residues (V158F), and thus we cannot measure directly how this polymorphism affects the binding affinity to the lower hinge region of IgG, directed against pneumococcal antigens. Therefore, because the HL-60 cells are always the same when performing the OPA, the FCGR3A genotype of the HL-60 cells is not expected to have an impact on the OPA results, as it affects the receptor and not the Fc region of IgG, which is patient-specific in the assay. Of note, the HL-60 cells express low levels of FcγRIII, which interact with the Fc region of IgG.

However, FcγR IIIa is also found in a soluble form. It may be assumed that the soluble FcγR IIIa could indirectly impact on antibody production, in line with a recent, comprehensive review [21]. FcγR IIIa was detected in human sera in a soluble form, resulting from proteolytic cleavage by matrix metalloproteinases upon activation of NK cells and neutrophils by protein antigens [21]. Soluble FcγRs presented immunomodulatory properties. For example, they inhibited B cell proliferation and IgM and IgG production [38]. Moreover, crosslinking of activating FcγRs such as FcγR IIIa led to the activation of some nuclear factors such as NFAT, which induces the expression of cytokines important for inflammation and immune regulation, e.g., IL-2, IL-6, IL-8, IL-10, TNF-α, and IFN-γ [40,41,42]. Finally, the only activating Fcγ receptor on NK cells is FcγRIIIa, which leads after activation to antibody-dependent, cell-mediated cytotoxicity (ADCC). Fcγ receptors expressed on many immune cells are thus capable of inhibiting and activating different cellular responses, which is important not only for controlling microbial infections, but also for regulating immunity [21,43,44,45]. Previous data show that the dimorphism in the FCGR3A gene affects the affinity to IgG2 and IgG4 [21]. Carriers of the V158 vs. F158 allele of FCGRIII3A showed approximately twofold higher affinity for IgG [17]. FcγR IIIa is expressed on NK cells, T cells, and subsets of monocytes and macrophages [46]. Because IgG2 and IgA antibody responses after pneumococcal vaccination correlated with FCGR3A genotypes in our study, one may speculate that the effects on antibody concentrations were indirect and most likely by immune regulation. It could be hypothesized that regulatory properties of soluble Fc receptors may be different in heterozygotes for the FCGR3A V158F polymorphism, i.e., they could inhibit the production of immunoglobulins to a higher extent or interact differently with other Fcγ and Fcα receptors, which may then also affect the concentration of IgA.

FcγRs are differentially expressed on immune cells and can be either activating or inhibitory, e.g., with FcγRIIa and FcγRIIIa belonging to the first group [43]. Data on FCGR3A and pneumococcal immunity are not yet published. However, the association of FCGR3A with vaccination-induced antibody responses may be stronger than with FCGR2A [1], as we observed consistent and significant results for FCGR3A. It was previously described that the course of infection with *S. pneumoniae* [1] or with *Plasmodium falciparum* [47] was influenced by the FCGR2A rs1801274 (FcγRIIa-H131R) polymorphism. Of note, IgG2 antibodies exclusively interact with FcγRIIa, and the interaction is mainly restricted to the FcγRIIa-H131 allele [48]. On the contrary, both alleles of FcγRIIa interact with IgG1 and IgG3 [48]. Children homozygous for FcγRIIa-R131 showed a significant increase in recurrence of acute otitis media after pneumococcal vaccination, which consisted of a 7-valent pneumococcal conjugate vaccine (Prevnar 7) and a 23-valent pneumococcal polysaccharide vaccine [1]. Less efficient interaction between pneumococcal antibodies and FcγRIIa was postulated, as the children did not differ in the concentrations of antibodies [1], similar to the current study. Data by the same group indicate that the effective phagocytosis of *S. pneumoniae* depends on FcγRIIa, with significantly higher phagocytosis levels in HH homozygotes compared to RR carriers [49,50]. In the current study, however, we did not observe an influence of FcγRIIa on functional antibody responses, as determined by OPA. The effect may be masked as vaccination led to an increase in (total) IgG, IgG2, and IgA antibodies [22], and the OPA measures all functionally active antibodies and not solely IgG2.

Furthermore, carriers of the AG genotype of FCGR3A showed, by trend, more IgG antibodies against HLA and MICA and inferior graft function. Of note, the presence of donor-specific IgG antibodies against HLA is an established risk factor for allograft survival [51]. Data on an intronic dimorphism in the FCGR3B gene were less clear. This dimorphism (also referred to as neutrophil antigen 1 vs. 2) affects N-linked glycosylation of the FcγR and, thereby, receptor function [17,19]. Whereas a study on 56 pediatric kidney transplant recipients did not observe a significant correlation between the FCGR3A and FCGR3B dimorphisms and allograft rejection [11], a study on 85 adult kidney transplant recipients with donor-specific HLA antibodies—who were recruited upon the antibody screening of 741 prevalent patients—found a higher rate (and extent) of peritubular capillaritis in protocol biopsies in homozygous and heterozygous carriers of FcγRIIIa-V158 (the FCGR3A genotypes AA and AG) [15]. NK92 cells expressing FcγRIIIa-V158 produced significantly more interferon-γ upon incubation with HLA antibody-coated cells than those expressing FcγRIIIa-F158 [15]. Moreover, it was described that the FcγRIIIa-F158V polymorphism influenced the efficacy of depleting antilymphocyte antibodies [52]. Thus, the protective effect of FcγRIIIa-F158 (the FCGR3A genotype GG) against rejection and allograft loss is in line with the previous literature [15]. Extending these previous data, we observed that especially heterozygous carriers of FcγRIIIa-V158 (the FCGR3A genotype AG) may be at risk of allograft rejection and loss. Furthermore, in accordance with that large previous study [15], the genotypes of FCGR2A and FCGR3B did not correlate significantly with allograft function.

Our study has several limitations. As the cohort was rather small, the results should be confirmed by larger independent studies. Specifically, the findings on the pneumococcal antibody ELISA need to be repeated. Moreover, we unfortunately have no information on the FCGR3A genotype of the HL-60 cell line used for the OPA. Genotyping for FCGR polymorphisms is thus one of the next steps. Finally, this study does not include an analysis of the potential mechanisms of how the polymorphism impacts the antibody concentration or function, which has to be further analyzed.

## 5. Conclusions

This study is the first showing that FCGR3A rs396991 (FcγRIIIa-V158F) impacts the efficacy of pneumococcal vaccination. A deeper understanding of this effect might help to optimize vaccination programs (e.g., the timing or an increased dose of the vaccine) in order to ensure a sufficient strength of immune responses, especially in sensitive groups such as transplant recipients.

## Figures and Tables

**Table 2:** Kidney allograft function, stratified by the FCGR2A and FCGR3A genotypes.

SNP	Genotype			Model ^1^	OR	*p*
FCGR2A rs1801274		**Rejection** **(*n* = 6)**	**No Rejection** **(*n* = 41)**			
	AA	1 (17%)	15 (37%)	Dominant	0.6	0.65
	AG	2 (33%)	15 (37%)	Recessive	0.4	0.34
	GG	3 (50%)	11 (27%)			
		**Graft Failure** **(*n* = 6)**	**No Graft Failure** **(*n* = 41)**			
	AA	2 (33%)	14 (34%)	Dominant	1.0	1.00
	AG	2 (33%)	15 (37%)	Recessive	0.8	1.00
	GG	2 (33%)	12 (29%)			
		**Graft Loss** **(*n* = 5)**	**No Graft Loss** **(*n* = 42)**			
	AA	0 (0%)	16 (38%)	Dominant	0.0	0.15
	AG	2 (40%)	15 (36%)	Recessive	0.2	0.15
	GG	3 (60%)	11 (26%)			
FCGR3A rs396991		**Rejection** **(*n* = 6)**	**No Rejection** **(*n* = 41)**			
	GG	0 (0%)	19 (46%)	Dominant	0.0	0.07
	AG	5 (83%)	17 (41%)	Recessive	0.7	1.00
	AA	1 (17%)	5 (12%)			
		**Graft Failure** **(*n* = 6)**	**No Graft Failure** **(*n* = 41)**			
	GG	1 (17%)	18 (44%)	Dominant	0.3	0.38
	AG	5 (83%)	17 (41%)	Recessive	Infinite	1.00
	AA	0 (0%)	6 (15%)			
		**Graft Loss** **(*n* = 5)**	**No Graft Loss** **(*n* = 42)**			
	GG	0 (0%)	19 (45%)	Dominant	0.0	0.07
	AG	4 (80%)	18 (43%)	Recessive	0.5	0.51
	AA	1 (20%)	5 (12%)			

Data were analyzed by Fisher’s exact test, using either a dominant or recessive model. ^1^ Test conditions: allele A (major allele) vs. allele B (minor allele); AA vs. AB plus BB (dominant model); AA plus AB vs. BB (recessive model). SNP—single nucleotide polymorphism; OR—odds ratio.

## Data Availability

The data presented in this study are available on request from the corresponding author. The data are not publicly available due to privacy restrictions.
